# Secondary thrombotic microangiopathy following lung transplantation: a multicenter European cohort study

**DOI:** 10.3389/ti.2026.16046

**Published:** 2026-08-05

**Authors:** Zsofia Rosselli, Jan Havlin, Berta Saez-Gimenez, François M. Carlier, Gabriella Muraközy, Peter Jaksch, Stefan Schwarz, Dominik Damm, Mace Schuurmans, Nicolas Kozakowski, Martina Gaggl, Alice Schmidt, Gere Sunder-Plassmann, Christof Aigner

**Affiliations:** 1 Department of Pulmonology, University Hospital of Zurich, Zurich, Switzerland; 2 Lung Transplant Program, Third Department of Surgery, First Faculty of Medicine, Charles University in Prague and Motol University Hospital, Prague, Czechia; 3 Lung Transplant Program, Department of Pulmonology Hospital Universitari Vall Hebron, Barcelona, Spain; 4 Pneumologie et Transplantation Pulmonaire, UCL Namur University Hospital | Godinne Site, Namur, Belgium; 5 Department of Thoracic Surgery, Medical University Vienna, Vienna, Austria; 6 Department of Pathology, Medical University Vienna, Vienna, Austria; 7 Division of Nephrology and Dialysis, Department of Medicine III, Medical University Vienna, Vienna, Austria; 8 Center for Public Health, Department of Social and Preventive Medicine, Medical University Vienna, Vienna, Austria

**Keywords:** clinical outcomes, European lung transplant centers, lung transplantation, multicenter cohort, secondary thrombotic microangiopathy

## Abstract

Secondary thrombotic microangiopathy (sTMA) is a rare but severe complication after lung transplantation. We performed a multicenter retrospective cohort study including adult lung transplant recipients diagnosed with *de novo* sTMA between 2010 and 2023 across five European transplant centers. The primary outcome was incidence of sTMA, with secondary outcomes including clinical presentation, treatment strategies, renal outcomes, and survival. Among 3,747 lung transplantations, 49 cases of sTMA were identified, corresponding to an incidence of 1.33%. Median time to diagnosis was 312 days (IQR 93–546), with 53% occurring within the first post-transplant year. All patients presented with acute kidney injury and laboratory signs of hemolysis, neurological complications were observed in 8%. Therapeutic approaches included plasma exchange (46.9%), anti-C5 therapy (24.5%), and sequential plasma exchange followed by complement inhibition (10.2%). At follow-up, 65.3% developed chronic kidney disease, 20.4% progressed to end-stage renal disease, and 10.2% died during the acute episode. No significant association between treatment modality and renal or survival outcomes was observed. sTMA after lung transplantation is infrequent but associated with substantial renal morbidity and mortality. Early recognition through assessment of hemolysis and kidney injury, supported by renal biopsy in selected cases, may facilitate timely diagnosis and management.

## Graphical Abstract

**Figure:**
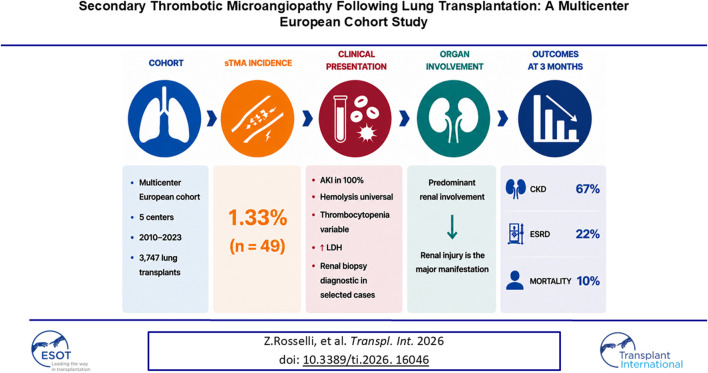

## Introduction

Thrombotic microangiopathy (TMA) is a rare but potentially life-threatening complication characterized by microvascular endothelial injury, leading to a triad of hemolytic anemia, thrombocytopenia, and organ damage, primarily affecting the kidneys and central nervous system [1].

Secondary thrombotic microangiopathy (sTMA) refers to TMA occurring in association with an identifiable trigger, such as transplantation, infection, malignancy, autoimmune disease, or drug exposure. Although complement activation may contribute to endothelial injury in selected cases, sTMA is generally distinguished from primary complement-mediated TMA by the presence of an underlying precipitating condition [2]. While sTMA has been extensively studied in these populations, its occurrence following lung transplantation (LuTX) remains poorly understood. Although some studies have estimated the incidence of sTMA in lung transplant recipients to be between 2% and 7%, the true prevalence and underlying mechanisms remain unclear [3–7].

Several risk factors for sTMA development after LuTX have been identified, including endothelial injury during surgery, ischemia-reperfusion injury, and the use of immunosuppressive agents such as calcineurin inhibitors and mTOR inhibitors. Despite these identified risks, the precise pathophysiology of sTMA in lung transplant recipients is still under investigation. Notably, not all cases of sTMA present with systemic hemolysis, which can make diagnosis challenging, particularly when renal injury occurs without the typical hematologic features. Furthermore, early identification and treatment options remain underexplored, with therapies such as plasmapheresis and complement inhibition showing mixed results in transplant populations [8–10], [10–12].

In recent years, a few small case series have shed light on the clinical characteristics and outcomes of sTMA following lung transplantation. However, these reports have been limited by small sample sizes and single-center designs. A recent study with approximately 80 patients represents one of the larger efforts to understand sTMA in LuTX recipients [13], yet this remains a rare and underexplored complication [4]. To date, no multicenter studies have systematically examined the incidence, risk factors, and clinical outcomes of sTMA after lung transplantation across different European centers.

This study aims to fill this gap by conducting a multicenter, retrospective cohort analysis of adult lung transplant recipients diagnosed with sTMA across five major European transplant centers. By evaluating the incidence, risk factors, treatment strategies, and renal outcomes in a larger cohort, we aim to provide more robust data that could inform diagnostic and therapeutic strategies for this rare but potentially severe complication of lung transplantation [4–6, 8–12, 14].

## Patients and methods

We conducted a multicenter, retrospective cohort study across five major European lung transplantation (LuTX) centers. The study included adult patients who underwent lung transplantation between 2010 and 2023 and were diagnosed with *de-novo* secondary thrombotic microangiopathy (sTMA).

### Inclusion and exclusion criteria

Adult patients (≥18 years) who underwent lung transplantation between 2010 and 2023 at the participating centers and developed *de-novo* secondary thrombotic microangiopathy (sTMA) were included in the study. Diagnosis of sTMA was based on clinical, laboratory, and histopathological criteria, with a focus on renal involvement. Diagnostic criteria included hemolysis, thrombocytopenia, organ damage, elevated lactate dehydrogenase (LDH) levels, reduced platelet counts, and ADAMTS13 activity (when available), with renal biopsy used to support diagnosis where available.

Exclusion criteria were: (1) patients with primary TMA (e.g., typical or atypical hemolytic uremic syndrome [HUS] or thrombotic thrombocytopenic purpura [TTP]) diagnosed before lung transplantation; (2) patients with incomplete data on transplant-related variables, post-transplant complications, or those lost to follow-up.

### Data collection

Data were collected from five major European lung transplantation centers between June and November 2024. A total of 20 centers were initially contacted via email and telephone to inquire about the diagnosis of secondary thrombotic microangiopathy (sTMA) in lung transplant recipients. Five centers provided anonymized case-level data, which were subsequently included in the analysis. A full list of the contacted centers and all definitions are available in Supplementary Material.

For each sTMA case, detailed clinical data were gathered using a standardized case report form, which was completed with the assistance of local transplant teams. The data collected included demographic information, transplant-related factors, immunosuppressive therapy details, infection history, and post-transplant complications (Supplementary Table S1).

### Study outcomes

The primary outcome of the study was the incidence of sTMA in lung transplant recipients across the participating centers. Secondary outcomes included, identification of risk factors associated with the development of sTMA, with particular focus on immunosuppressive regimens, infection history, and post-transplant complications. Clinical outcomes following diagnosis, including progression to chronic kidney disease (CKD), end-stage renal disease (ESRD), and mortality after the end of the TMA episode (i.e., hospital discharge, normalization of laboratory parameters, death) or after 3 months, whichever came first. Evaluation of the impact of different immunosuppressive therapies on the onset and progression of sTMA. Assessment of renal recovery following the TMA episode, as well as the presence of neurological involvement at presentation.

### Statistical analysis

Descriptive statistics were used to summarize baseline characteristics, transplant-related variables, and clinical features at the time of sTMA diagnosis. Continuous variables are presented as mean ± standard deviation (SD) or median with interquartile range (IQR), depending on data distribution, while categorical variables are reported as absolute numbers and percentages.

Chi-square tests were used to compare categorical variables between groups.

The primary outcome was a composite endpoint defined as chronic kidney disease (CKD) stage 5 or death at 3 months, dichotomized as poor outcome (CKD stage 5 or death) versus favourable outcome (CKD stages 1–4).

To assess associations between clinical variables and the primary outcome, binary, multivariate logistic regression was performed. Covariates included age, sex, KDIGO acute kidney injury (AKI), requirement of renal replacement therapy during the index episode, and eculizumab treatment status.

Results are reported as odds ratios (OR) with corresponding 95% confidence intervals (CI). Statistical significance was defined as a two-sided p-value <0.05.

All statistical analyses were conducted using JASP 0.96, and a two-sided p-value of <0.05 was considered statistically significant.

### Ethical considerations

The study was approved by the Ethics Committee of the Medical University of Vienna (reference number 1361/2014). All procedures were carried out in accordance with the STROBE guidelines for observational research.^1^

## Results

**FIGURE 1:**
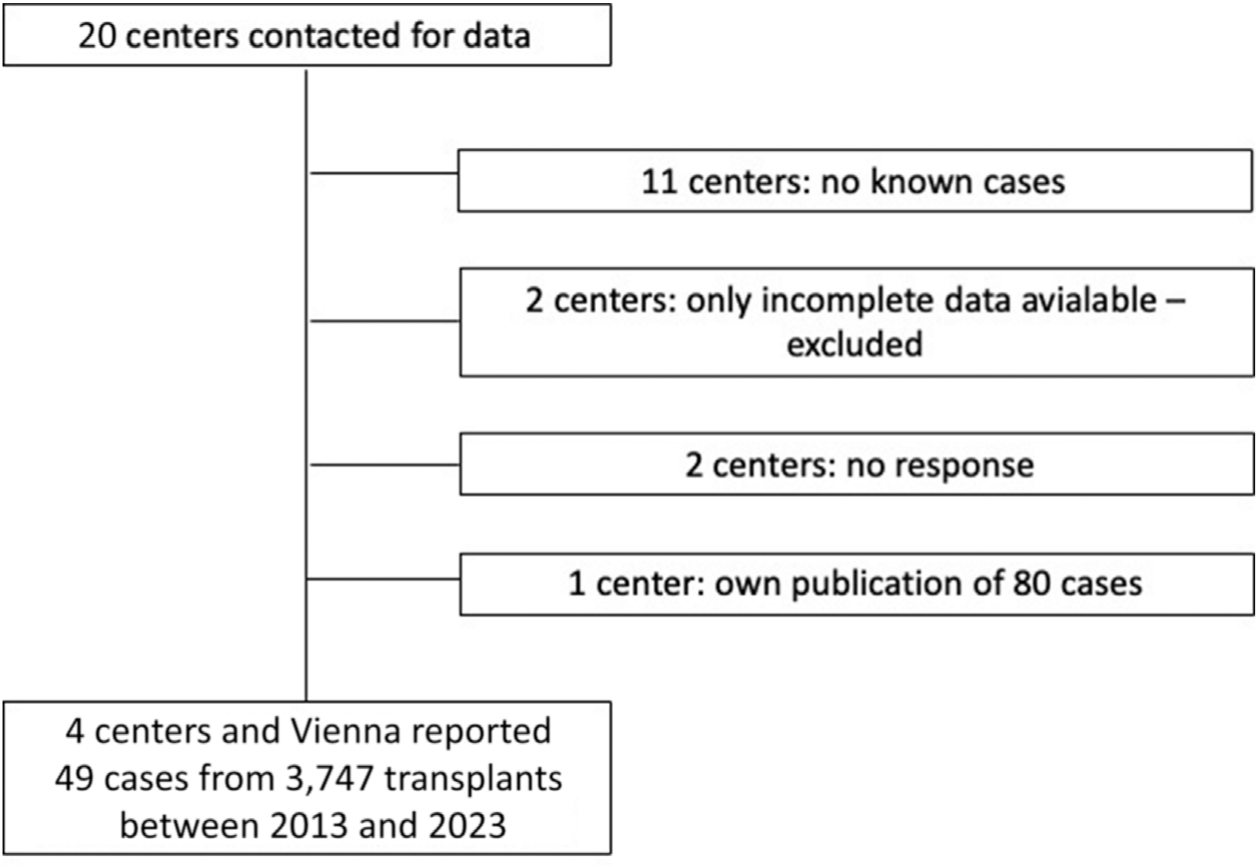
Flow chart on center disposition.

A total of 49 patients with a diagnosis of secondary thrombotic microangiopathy (sTMA) were identified across the participating centers during the observation period (Table 1). During the study period, 3,747 lung transplants were performed across the five reporting centers, yielding an incidence of sTMA of 1.33% among lung transplant recipients (Figure 1, Supplementary Material).

**TABLE 1:** Patient characteristics.

Characteristic	All patients
Patient number, n	49
Female, n	24 (49%)
Mean age at transplant, years	54 (±11.2)
Median time from TX to TMA, days	312 (IQR 93–546)
CMV high risk, n	13 (27%)
Lung disease leading to transplant	
COPD, n	27 (55%)
ILD/fibrosis, n	12 (24%)
Cystic fibrosis, n	4 (8%)
Alpha 1 AT, n	3 (6%)
Other, n	3 (6%)
Induction therapy	
Alemtuzumab, n	15 (30.6%)
Anti-thymocyte globulin, n	11 (22.4%)
Basiliximab, n	10 (20.4%)
None, n	8 (16.3%)
Unknown, n	5 (10.2%)
Immunosuppression regimen	
Tacrolimus-based, n	33 (67%)
Cyclosporine-based, n	13 (26%)
Tacrolimus + mTOR inhibitor, n	3 (6%)*

Abbreviations: anti C5 treatment, eculizumab or ravulizumab; TX, transplantation; TMA, thrombotic microangiopathy; CMV; cytomegalovirus; COPD, chronic obstructive pulmonary disease; ILD, interstitial lung disease; AT, antitrypsin; mTOR, mammalian target of rapamycin.

* Included in tacrolimus-based group.

The cohort consisted of 24 females (49%), with a mean age of 54 years at the time of transplantation. The most common underlying diseases leading to lung transplantation were chronic obstructive pulmonary disease (COPD) (55%), interstitial lung disease (ILD) (24%), cystic fibrosis (8%), and alpha-1-antitrypsin deficiency (6%). Pre-transplant renal disease history was unavailable for most of the cohort. The median time from lung transplantation to sTMA diagnosis was 312 days (IQR 93–546). 11 patients (22.4%) were diagnosed within the first month post-transplant, and 15 patients (30.6%) were diagnosed within the first year. In total, 26 patients (53%) were diagnosed within the first year following transplantation. Cytomegalovirus (CMV) disease was present in 6 patients (12.2%) at the time of sTMA diagnosis.

For induction immunosuppressive therapy 15 patients (30.6%) received alemtuzumab, 11 patients (22.4%) received anti-thymocyte globulin, 10 patients (20.4%) received basiliximab and 8 patients (16.3%) did not receive induction therapy, and data were missing for 5 patients (10.2%).

Pre-transplant sensitization was documented in 8 patients (16.3%), and a positive crossmatch was observed in 6 patients (12.2%), with missing data for 4 and 3 patients, respectively. Persistent donor-specific antibodies were detected in 11 patients (22.4%) at 6 months post-transplantation. Immunosuppressive regimens included tacrolimus-based therapy in 73% of patients and cyclosporine-based therapy in 27%. Chronic lung allograft dysfunction (CLAD) was diagnosed in 16 patients (32.7%), with a median time to CLAD onset of 549 days post-transplant.

Four patients (8%) developed antibody-mediated rejection (AMR), with two occurring prior to and two following sTMA episodes, all within the first-year post-transplant. Additionally, six patients experienced acute cellular rejection (ACR).

### TMA episode characteristics

At the time of diagnosis, acute kidney injury (AKI) was present in all patients (Table 2). The mean hemoglobin concentration was 8.9 ± 1.6 g/dL, the mean platelet count was 74 ± 56 G/L, and the mean lactate dehydrogenase (LDH) level was 1243 ± 809 IU/mL. The mean serum creatinine level was 2.87 ± 1.34 mg/dL. Acute kidney injury KDIGO stage 1, 2 and 3 were present in 20 (40.8%) patients, 5 (10.2%) patients and 24 (49%) patients, respectively. A total of 23 patients (46.9%) received RRT during the episode and of those 10 (43%) remained dependent on chronic RRT and 3 (13%) died during the episode. The other 11 patients had a mean eGFR of 45 (±17) mL/min/1.73 m^2^ 3 months after the TMA episode. Mean serum creatinine before the beginning of the TMA episode was 1.14 mg/dL (±0.58). After 3 months 11 (22.4%) patients had CKD stage 4 or lower, 5 (10.2%) were dependent on chronic RRT and 5 (10.2%) had died.

**TABLE 2:** Presentation and outcome of sTMA.

Characteristic	All patients
Mean laboratory parameter at presentation	
Hemoglobin (g/dL)	8.91 (±1.58)
Platelets (G/L)	74 (±56)
Lactate dehydrogenase (U/L)	1243 (±809)
ADAMTS13 (% activity)	68.96 (±24.94)
Serum creatinine (mg/dL)	2.87 (±1.34)
KDIGO AKI stages	
Stage 1	20 (40.8%)
Stage 2	5 (10.2%)
Stage 3	24 (49%)
RRT during TMA episode	23 (46.9%)
Presumed CMV disease at TMA diagnosis, n	6 (12%)
Kidney biopsy available, n	11 (22.4%)
Therapy after TMA diagnosis	
Switch of IS regimen, n	41 (83.7%)
Plasma therapy at diagnosis, n	23 (46.9%)
Anti C5 treatment, n	12 (24.5%)
First plasma, then anti c5, n	5 (10.2%)
Outcome of TMA episode	
CKD, n 33	33 (67.3%)
ESRD, n	11 (22.4%)
Death, n	5 (10.2%)
ESRD after supportive treatment, n	2 (4.1%)
ESRD after treatment with PEX, n	3 (6%)
ESRD after treatment with PEX then Ecu, n	3 (6%)
ESRD after treatment with Ecu, n	2 (4.1%)
Multiple TMA episodes, n	2 (4.1%)
Genetic investigations	
CFH/CFI/CD46, n	20 (40.8%)
All relevant genes, n	10 (20.4%)
n/a, n	29 (59.2%)
Rare genetic variants, n	5 (10%)

Abbreviations: ADAMTS13, A Disintegrin and Metalloproteinase with Thrombospondin Motif 13; eGFR, estimated glomerular filtration rate; CMV, cytomegalovirus; TMA, thrombotic microangiopathy; IS, immunosuppression; anti C5 treatment, eculizumab or ravulizumab; CKD, chronic kidney disease; ESRD, end stage renal disease; PEX, plasma exchange; ecu, eculizumab; CFH, complement factor H; CFI; complement factor I; n/a, not available).

**FIGURE 2:**
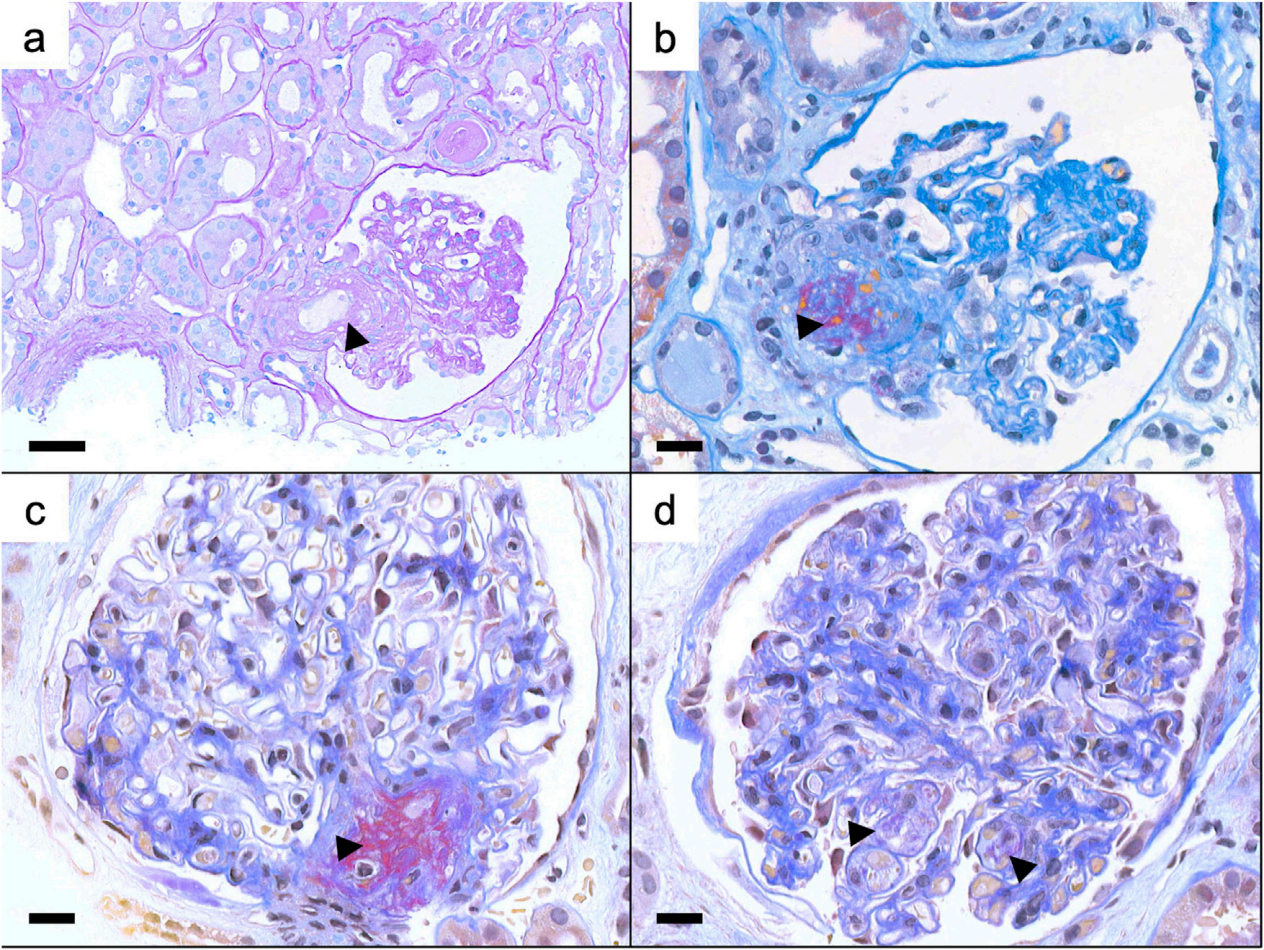
Histological TMA after LuTX in kidney biopsies. **(A)** Glomerulus with collapsed capillary loops, intimal mucoid changes, and vacuolation of smooth muscle cells (black arrowhead) at the vascular pole (Periodic Acid-Schiff stain (PAS) stain; scale bar = 50 µm). **(B)** Deeper section of the same collapsed glomerulus, showing a thrombus composed of fibrin and erythrocytes (black arrowhead) at the vascular pole (Acid Fuchsin Orange G (AFOG) stain; scale bar = 20 µm). **(C)** Another glomerulus with polar thrombosis (fibrin, black arrowhead) extending toward nearby glomerular capillaries (AFOG stain; scale bar = 20 µm). **(D)** Glomerulus with global endothelial cell swelling and segmental thromboses (black arrowheads) containing some fibrin, and subendothelial or intramural debris of erythrocytes or platelet (AFOG stain; scale bar = 20 µm).

Neurological complications were noted in 4 patients (8%). ADAMTS13 activity was measured in 86% of patients, with a mean level of 69% ± 25%. Haptoglobin levels were undetectable in all patients. Renal biopsy was performed in 10 patients (20.4%), and all biopsies demonstrated typical features of acute TMA like fibrin thrombi and endothelial cell activation. Chronic TMA lesions (double contouring of basal membranes and multilayering of arteries and arterioles) were only seen in 1 patient (Figure 2).

Upon diagnosis, 23 patients were treated with plasma exchange (46.9%), another 5 were first started with plasma exchange and then switched to eculizumab (10.2%) and 12 (24.5%) were treated with eculizumab as a first line therapy. Median time from diagnosis to first plasma exchange was 1 day (range 0–246 days) and 2 days (range 0–232 days) from diagnosis to first eculizumab. Of the 24 patients with AKI stage 3, 3 (12.5%) received supportive therapy, 9 received plasma exchange (37.5%), 8 (33.3%) received eculizumab and 4 received eculizumab after plasma exchange (16.7%). In those 4 patients time from first plasma exchange to first eculizumab was 2 days in one patient and 21 days in the three others.

In a multivariable logistic regression model, age was independently associated with the composite outcome of CKD stage 5 or death at 3 months (OR 1.07, p = 0.012). Eculizumab treatment was not significantly associated with the composite outcome of CKD stage 5 or death at 3 months (OR 0.73, 95% CI 0.12–4.61, p = 0.74 Supplementary Table S2).

Modifications to immunosuppressive regimens were made in 83.6% (n = 41) of patients. The most common modification was switching from tacrolimus to cyclosporine (n = 35, 66%). Other changes included switching to belatacept (n = 3, 5.7%), mTOR inhibitors (n = 2, 4.1%), or MMF (n = 1, 2%).

### Renal and clinical outcomes

**FIGURE 3:**
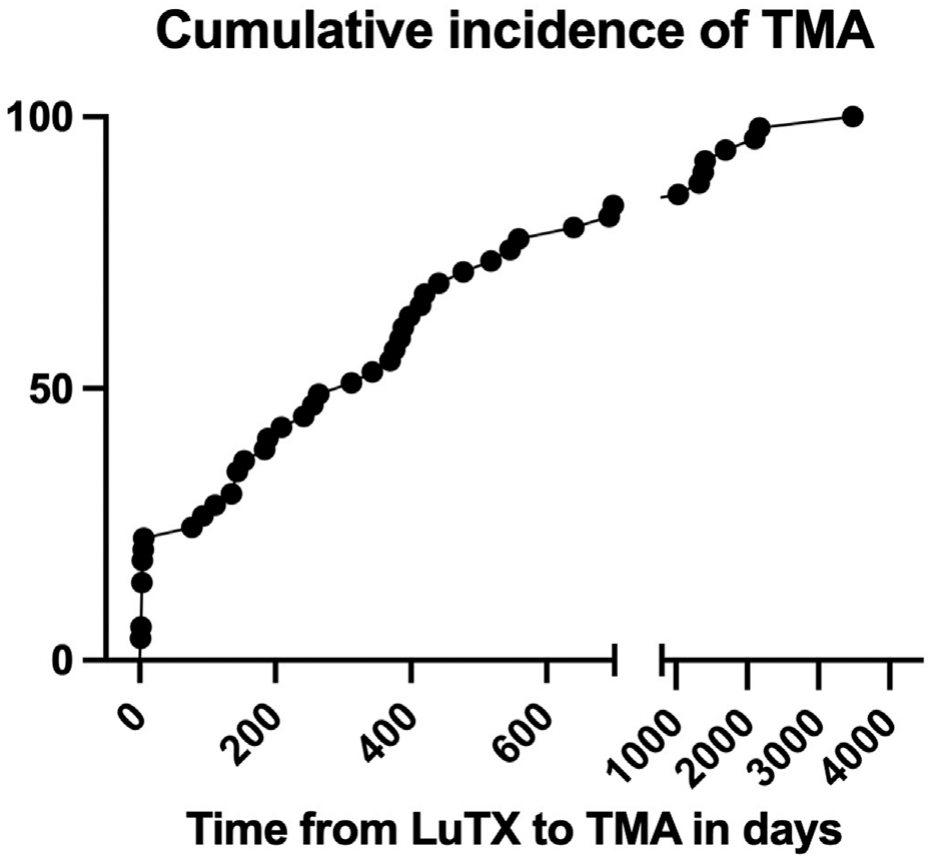
Cumulative incidence of secondary thrombotic microangiopathy (TMA) after lung transplantation. X-axis: Time from lung transplantation (days). Y-axis: Cumulative incidence of secondary thrombotic microangiopathy (TMA).

At 3 months follow-up chronic kidney disease (CKD) of any grade developed in 33 patients (67.3%), end-stage renal disease (ESRD) was observed in 11 patients (22.4%) and 5 patients (10.2%) died during the acute episode of sTMA, with one patient being dialysis-dependent prior to sTMA diagnosis (Figure 3).

Genetic investigations were only performed in a minority of patients and rare variants were found in 5 (10%) of patients, with one variant in CFH considered clinically significant and the other variants were considered to be of unknown significance.

## Discussion

Secondary thrombotic microangiopathy (sTMA) is increasingly recognized as a serious complication following solid organ transplantation, though it remains underreported, particularly after lung transplantation. In this study, we observed an incidence of sTMA of 1.33% of 3,747 lung transplants performed across the participating centers. This is consistent with a study from France, which reported an incidence of 1.8% among 4,565 lung and heart-lung transplant recipients [14]. While our results align with these findings, our incidence is lower than previously reported in smaller studies, such as the 2.9% incidence reported by Rosseels et al. and the 3.8 cases per 100 patient-years seen in Hachem et al.’s study [4, 6]. This discrepancy could be due to variations in diagnostic criteria, immunosuppressive protocols, or underreporting of sTMA, underscoring the need for standardized diagnostic approaches across centers.

We observed significant intercenter variability in the recognition and documentation of sTMA, with 11 out of 20 centers reporting no cases. This highlights the importance of uniform diagnostic criteria and suggests that diagnostic thresholds for sTMA may vary considerably. These variations may contribute to discrepancies in the reported incidence and underscore the necessity of improving consistency in TMA reporting across transplant centers. Renal involvement was nearly universal in our cohort, with hemolysis (indicated by undetectable haptoglobin levels) observed in all patients, confirming that renal injury is a core feature of sTMA. Thrombocytopenia and elevated lactate dehydrogenase (LDH) were present in a subset of patients but not consistently, making diagnosis challenging in some cases. In our cohort, renal biopsy was performed in 10 patients (20.4%), and it was diagnostic in all cases, emphasizing the importance of kidney biopsy in confirming the diagnosis of sTMA when clinical suspicion is high but hematologic signs are not fully consistent.

The baseline characteristics of our cohort reflect the typical European lung transplant population, with a mean age of 54 years and a predominance of chronic obstructive pulmonary disease (55%), interstitial lung disease (24%), and cystic fibrosis (8%). The high prevalence of pre-transplant sensitization (16.3%) and positive crossmatch (12.2%) further underscore the immunological burden seen in lung transplant recipients, which may predispose them to the development of complications such as sTMA. Notably, CMV high-risk status was observed in 27% of our cohort, and 12% of patients developed sTMA in the context of presumed CMV reactivation. This suggests that CMV reactivation, combined with intensified immunosuppressive therapy, may act synergistically to trigger sTMA, as seen in other transplant populations. However, the exact pathophysiological mechanisms remain speculative, and further investigation is needed to better understand these interactions. Interestingly there are 2 timepoints when *de novo* TMA after LuTX occurs. Firstly, in the first few days after the transplant with 22% of the cohort diagnosed in the first thirty days. Then after 100 days on, when infectious complications like CMV are more common. Genetic investigations in a cohort of secondary TMA remains a topic of debate. In recent literature rare variants are reported to be found in as many as 30% of patients, while other larger cohort report variants in only 5% of patients [11, 12]. Since no single, definitive marker to distinguish complement-mediated TMA from secondary TMA is available, one can speculate that the distinction between the disease is not always flawless.

Treatment strategies for sTMA in our cohort were highly variable. Plasma exchange was performed in 55.1% of patients, while eculizumab was used in 26.5% of patients as first-line therapy and in 10.2% after plasma exchange. Despite the variability in treatment strategies, no significant difference in outcomes (progression to end-stage renal disease (ESRD), or mortality) was found between the treatment groups. This lack of difference in outcomes is consistent with previous studies that found immunosuppressive modifications, including switching calcineurin inhibitors or adding mTOR inhibitors, often fail to prevent recurrent episodes of sTMA [14].

Eculizumab has been used in secondary TMAs in various case reports and case series [14, 15]. Earlier administration of eculizumab has been associated with better outcomes [5, 16]. This highlights the importance of early recognition and prompt therapeutic intervention in preventing severe renal complications. In our cohort despite early treatment initiation eculizumab was not associated with a more favorable outcome. However, this might be due to our low event rate and relatively small sample size. Further studies are needed to assess the optimal timing and effectiveness of eculizumab in lung transplant recipients with sTMA.

This study has several limitations. Its retrospective, multicenter design introduces the possibility of underreporting and missing data, particularly regarding pre-transplant renal function and ADAMTS13 testing. Case identification likely varied across centers, with lower-volume sites potentially underrecognizing or underreporting sTMA, which may have led to underestimation of true incidence. Treatment strategies, including plasma exchange, eculizumab, and immunosuppressive modifications, were not standardized and depended on local protocols, physician judgment, and disease severity, introducing potential indication bias and inter-center variability. Kidney biopsy was performed in only a subset of patients, limiting assessment of acute versus chronic lesions and their impact on outcomes. Finally, patient-level data for all lung transplant recipients were unavailable, precluding analyses of lung graft survival or time-dependent outcomes beyond the defined 3-month post-TMA episode. Despite these limitations, our findings provide the largest multicenter assessment to date of post-lung transplant sTMA and underscore the need for prospective, standardized studies to optimize diagnosis, risk stratification, and management.

Secondary thrombotic microangiopathy following lung transplantation, though rare, is associated with significant renal morbidity and mortality. Early recognition and intervention are crucial, particularly in patients presenting with acute kidney injury and hemolysis. Clinicians need to be aware of this complication and centers should adopt a standard of practice for diagnosis and treatment. Renal biopsy remains a valuable diagnostic tool in uncertain cases. Although treatment strategies varied, early initiation of targeted therapy with eculizumab may offer a more effective approach, though further studies are needed to confirm its efficacy. Standardized diagnostic criteria and treatment protocols, along with earlier intervention, could substantially improve outcomes for lung transplant recipients affected by sTMA.

## Data Availability

The datasets presented in this study can be found in online repositories. The names of the repository/repositories and accession number(s) can be found in the article/Supplementary Material.

## Footnotes

1
http://www.strobe-statement.org/
