# Supplementary material for: Secondary thrombotic microangiopathy following lung transplantation: a multicenter European cohort study
Source: Transpl Int. 2026 Aug 5;39:16046. doi: 10.3389/ti.2026.16046 (PMC13485827; doi:10.3389/ti.2026.16046)
Supplement: Supplementary file 1 [file Supplementaryfile1.docx]

**Supplemental Material**

**Secondary Thrombotic Microangiopathy After Lung Transplantation: A Multicenter Survey Across European Transplant Centers**

Zsofia Rosselli^1,2^, Jan Havlin^4^, Berta Saez-Gimenez^5^, François Carlier^6^ Gabriella Muraközy^1^, Peter Jaksch^1^, Dominik Damm^2^, Mace Schuurmans^2^, Alice Schmidt^3^, Gere Sunder-Plassmann^3^, , Christof Aigner^3^

^1^	Department of Thoracic Surgery, Medical University Vienna, Vienna, Austria

^2^	Department of Pulmonology, University Hospital of Zurich, Zurich, Switzerland

^3^	Division of Nephrology and Dialysis, Department of Medicine III, Medical University Vienna, Vienna, Austria

^4^	Medical Director, Prague Lung Transplant Program Assistant Professor of Medicine,

Charles University Motol University Hospital, Praha, Czech Republic

^5^	Respiratory Department Hospital Universitari Vall Hebron. Barcelona, Spain

^6^	Pneumologie et Transplantation pulmonaire, UCL Namur University Hospital | Godinne Site, Namur, Belgium

**Figures with Legends**

**Supplementary Table S1. Variables Collected for Each Case of Secondary TMA Following Lung Transplantation**

| Category | Variable | Description |
| --- | --- | --- |
| Patient Information | ID | Anonymized patient identifier |
|  | Date of Birth | DD/MM/YYYY |
|  | Gender | Male/Female |
|  | Blood Group | ABO typing |
|  | Diagnosis | Indication for lung transplant |
|  | Transplant Date | DD/MM/YYYY |
|  | Type of Transplant | Single or Double lung |
| Transplant & Immunosuppression | Induction | Agents used (e.g., ATG, basiliximab) |
|  | Maintenance Immunosuppression | CNI, mTOR inhibitors, MMF, steroids, etc. |
|  | Immunosuppression switch | 1 = No, 2 = Cyclosporin, 3 = Belatacept, 4 = Other (specify) |
|  | Persistent DSA | 1 = Yes, 0 = No (DSA > 6 months) |
|  | Crossmatch | Virtual or CDC |
| Infection | Bacterial/Fungal Infection | Date of diagnosis |
|  | CMV Donor | CMV status (positive/negative) |
|  | CMV Recipient | CMV status (positive/negative) |
|  | CMV Viremia | Date >1000 copies/mL |
| Postoperative Course | Complications | 1 = Bleeding, 2 = Prolonged ECMO, 3 = Cardiovascular, 4 = Other (specify) |
| Rejection & CLAD | ACR | Histological grade and date |
|  | AMR | Date of diagnosis |
|  | CLAD | Date of diagnosis |
| TMA Episode | Diagnosis Date | Date of secondary TMA diagnosis |
|  | Neurologic Symptoms | 1 = Yes, 0 = No |
|  | Serum Creatinine | At presentation (mg/dL) |
|  | eGFR | CKD-EPI at presentation |
|  | Platelet Count | At presentation (G/L) |
|  | LDH | At presentation |
|  | Hemoglobin | At presentation |
|  | ADAMTS13 | At presentation |
|  | Disease-Causing Mutation | If applicable |
|  | Kidney Biopsy | 1 = Yes, 0 = No |
|  | Biopsy Result | Histopathologic findings |
| Treatment & Outcome | Therapy | 0 = Supportive, 1 = PE, 2 = Eculizumab, 3 = PE then ECU, 4 = Other (specify) |
|  | First PE | Date |
|  | First Eculizumab | Date |
|  | Outcome of Episode | 0 = CKD, 1 = ESRD, 2 = Death |
|  | Death Date | If applicable |
|  | Cause of Death | If applicable |
|  | Last Visit | Date of last follow-up |
| Center Info | Center | Name of reporting center |
|  | Total Transplants (n) | Number of lung transplants at the center (2010–2023) |

**Supplementary Table S2. Results of binary, multivariate logistic regression.**

| **Variable** | **OR** | **95% CI** | **p** |
| --- | --- | --- | --- |
| Age | 1.07 | 1.01–1.14 | 0.02 |
| Eculizumab | 0.73 | 0.12–4.61 | 0.74 |

Abbreviations: OR, odds ratio; CI, confidence interval.

*Responses were received from the following centers:

1. Unité de Transplantation Pulmonaire, Service de Pneumologie, Lausanne, Switzerland
2. Servicio de Cirugía Torácica y Trasplante Pulmonar, Hospital Universitario 12 de Octubre, Madrid, Spain
3. Hôpital Marie-Lannelongue, Le Plessis-Robinson, France – reported 80 cases (own publication)
4. Prague Lung Transplant Program, Motol University Hospital, Prague, Czech Republic – reported 13 cases
5. Tartu University Hospital, Tartu, Estonia
6. Hospital Universitario Marqués de Valdecilla, Santander, Spain
7. Semmelweis University, Budapest, Hungary
8. Royal Papworth Hospital NHS Foundation Trust, Cambridge, UK
9. King Faisal Specialist Hospital and Research Centre, Riyadh, Saudi Arabia
10. Klinika Chirurgii Klatki Piersiowej i Transplantacji PUM, Szczecin, Poland
11. National Danish Lung Transplant Program, Rigshospitalet, Copenhagen, Denmark
12. University Hospital Puerta de Hierro - Majadahonda, Madrid, Spain
13. Universitätsklinikum des Saarlandes, Homburg, Germany
14. Westdeutsches Herzzentrum Essen, Essen, Germany
15. CHU UCL Namur | Site Godinne, Namur, Belgium – reported 6 cases
16. University of Zurich, Zurich, Switzerland – reported 2 cases
17. Sahlgrenska University Hospital, Göteborg, Sweden
18. Universitätsklinikum Freiburg, Klinik für Thoraxchirurgie, Freiburg, Germany
19. Barcelona Center Vall d’Hebron, Barcelona, Spain – reported 6 cases
20. Great Ormond Street Hospital, London, UK

Responses were categorized as follows:

- **11 centers** reported no known cases of secondary TMA following lung transplantation.
- **2 centers** were unable to provide case information due to missing or incomplete documentation.
- **2 centers** did not respond to repeated contact attempts.
- **1 center** referred to their previously published a cohort (3)
- **4 centers** (Prague, Namur, Zurich, and Barcelona) reported a total of 27 individual cases of secondary TMA, which were shared for inclusion in this study.
  - **Prague: 516 transplants – 13 TMA cases**
  - **Namur: 334 transplants – 6 TMA cases**
  - **Barcelona:1125 transplants – 6 TMA cases**
  - **Zurich: 368 transplants – 1 TMA case**
  - **Vienna: 1404 transplants – 23 TMA cases**

**Definitions**

**Acute Cellular Rejection (ACR)** was defined based on histopathological evaluation of transbronchial lung biopsies, according to the International Society for Heart and Lung Transplantation (ISHLT) grading system. ACR was diagnosed when biopsy findings met criteria for perivascular and/or interstitial mononuclear cell infiltrates (grade A1 or higher), in the absence of infection.

**Antibody-Mediated Rejection (AMR)** was defined according to ISHLT criteria, requiring both histological findings consistent with AMR (e.g., capillaritis or neutrophilic margination) and immunopathologic evidence of complement activation (e.g., C4d deposition), in addition to the presence of circulating donor-specific antibodies (DSAs).^1^

**Chronic Lung Allograft Dysfunction (CLAD)** was defined as a sustained decline in forced expiratory volume in 1 second (FEV₁) of ≥20% from baseline (post-transplant best), persisting for more than 3 months, and not attributable to acute rejection, infection, or other identifiable causes. CLAD was diagnosed by two experienced transplant pulmonologists according to ISHLT guidelines. Phenotypes such as bronchiolitis obliterans syndrome (BOS) or restrictive allograft syndrome (RAS) were classified according to established guidelines. ).^2^

**Donor-specific antibodies (DSA)** Donor-specific antibodies (DSA) were detected using Luminex single-antigen bead technology. The de novo development of DSA was defined as the new appearance of HLA-specific antibodies against donor antigens at any time after transplantation. Patients were considered highly sensitized if they had a panel reactive antibody (PRA) level of 50% or greater prior to transplantation.

**Bacterial and fungal infections** were defined as microbiologically confirmed infections, either from sterile site cultures (e.g., blood, BAL fluid) or clinically relevant specimens, accompanied by compatible clinical symptoms and/or radiologic findings, and requiring antimicrobial therapy.

**KDIGO-AKI^3^**

Staging of AKI

Stage	Serum creatinine	Urine output

1	1.5–1.9 times baseline	<0.5 ml/kg/h for 6–12 hours

OR 0.3 mg/dl increase

2	2.0–2.9 times baseline	<0.5 ml/kg/h for $\geq$12 hours

3	3.0 times baseline	<0.3ml/kg/h for $\geq$ 24hours

OR	OR

Increase in serum creatinine to	Anuria for $\geq$ 12 hours

$\geq$4.0 mg/dl

OR

Initiation of renal replacement therapy

**Definitions**

CLAD was diagnosed by two experienced transplant pulmonologists according to ISHLT guidelines. Acute cellular and humoral rejection were classified according to ISHLT criteria. (17-19)

DSA was detected using Luminex-based single-antigen bead assays, and de novo DSA was defined as new onset after transplantation. A mean fluorescence intensity (MFI) >1000 was considered positive. Routine monitoring was performed at 1-, 3-, 6-, and 12-months post-transplant and in case of clinical suspicion.

Cytomegalovirus (CMV) viremia was assessed by PCR, with a viral load >1,000 copies/mL considered positive in line with institutional protocols and international guidelines.

**Literature**

1.	Levine DJ, Glanville AR, Aboyoun C, et al. Antibody-mediated rejection of the lung: A consensus report of the International Society for Heart and Lung Transplantation. *J Heart Lung Transplant* 2016; **35**(4): 397-406.

2.	Verleden GM, Glanville AR, Lease ED, et al. Chronic lung allograft dysfunction: Definition, diagnostic criteria, and approaches to treatment-A consensus report from the Pulmonary Council of the ISHLT. *J Heart Lung Transplant* 2019; **38**(5): 493-503.

3.	Khwaja A. KDIGO clinical practice guidelines for acute kidney injury. *Nephron Clin Pract* 2012; **120**(4): c179-84.
